# FLIP (Flice-like inhibitory protein) suppresses cytoplasmic double-stranded-RNA-induced apoptosis and NF-κB and IRF3-mediated signaling

**DOI:** 10.1186/1478-811X-9-16

**Published:** 2011-06-02

**Authors:** Priya Handa, Joan C Tupper, Katherine C Jordan, John M Harlan

**Affiliations:** 1 Division of Cardiology, Department of Medicine, Box 358055, 815 Mercer Street, University of Washington, Seattle, WA 98109, USA; 2 Diabetes and Obesity Center of Excellence, Department of Medicine, Box 358055, 815 Mercer Street, University of Washington, Seattle, WA 98109, USA; 3 Division of Hematology, University of Washington, Harborview Medical Center, Box 359756, 325 Ninth Avenue, Seattle, WA 98104, USA

## Abstract

**Background:**

Cytoplasmic viral double-stranded RNA (dsRNA) is detected by a class of ubiquitous cytoplasmic RNA helicases, retinoic acid inducible gene-I (RIG-I) and melanoma differentiation antigen-5 (MDA5), which initiate a signaling cascade via their common adaptor called interferon-β (IFN-β) promoter stimulator-1 (IPS-1). This leads to the production of proinflammatory and antiviral cytokines, the type I Interferons, via mainly nuclear factor kappa B (NF-κB) and interferon response factor-3 (IRF3) transcription factors. Fas-associated death domain (FADD) protein, receptor-interacting protein (RIP1), caspase-8 and tumor necrosis factor receptor (TNFR)-associated death domain (TRADD) protein, all traditionally associated with death receptor signaling, are also involved in RIG-I/MDA5 signaling pathway. We previously showed that FLIP (Flice-like inhibitory protein), also designated as *cflar *(CASP8 and FADD-like apoptosis regulator), negatively regulates lipopolysaccharide (LPS)-induced toll-like receptor 4 (TLR4) signaling in endothelial cells and mouse embryonic fibroblasts (MEFs) and protected against TLR4-mediated apoptosis.

**Results:**

In this study, we investigated the role of FLIP in cellular response to cytoplasmic polyinosinic:polycytidylic acid, poly(I:C), a synthetic analog of dsRNA. *C*onsistent with the previously described role of FADD in RIG-I/MDA5-mediated apoptosis, we found that FLIP^-/- ^MEFs were more susceptible to killing by cytoplasmic poly(I:C). However, FLIP^-/- ^MEFs also exhibited markedly increased expression of NF-κB-and IRF3- dependent genes in response to cytoplasmic poly(I:C). Importantly, reconstitution of FLIP in FLIP^-/-^MEFs reversed the hyper-activation of IRF3- and NF-κB-mediated gene expression. Further, we found that caspase-8 catalytic activity was not required for cytoplasmic poly(I:C)-mediated NF-κB and IRF3 signaling.

**Conclusions:**

These results provide evidence for a crucial dual role for FLIP in antiviral responses to cytoplasmic dsRNA: it protects from cytoplasmic dsRNA-mediated cell death while down-regulating IRF3-and NF-κB-mediated gene expression. Since the pathogenesis of several viral infections involves a heightened and dysregulated cytokine response, a possible therapy could involve modulating FLIP levels.

## Background

Cells respond to a viral challenge by rapidly producing type I Interferons (IFNs). The type I IFNs, IFN-α and IFN-β, are key cytokines, which induce an antiviral state and facilitate innate and adaptive immune responses [1]. The induction of IFN-β is regulated by several transcription factors such as nuclear factor kappa (NF-κB) and interferon regulatory factor-3 (IRF3). IRF3 activation requires phosphorylation by two kinases, TANK-binding kinase 1 (TBK1) and IκB kinase (IKK)ε [2]. Activated NF-κB and IRF3 translocate to the nucleus and trigger the expression of Type I IFNs, which are then secreted and bind to their cognate receptors on host cells. The mammalian Toll-like receptors (TLRs) that recognize the viral nucleic acids are TLR3, TLR7/8, and TLR9 for double-stranded RNA (dsRNA), single-stranded RNA, and DNA, respectively [1]. In addition, dsRNA is sensed by a ubiquitous family of cytoplasmic RNA helicases, retinoic acid-inducible gene-I (RIG-I) and Melanoma differentiation-associated gene (MDA-5) [3], jointly referred to as RIG-like helicases (RLHs). While both serve as cytoplasmic sensors of RNA and function through a common adaptor protein called IPS-1 (IFN-β promoter stimulator-1, also known as MAVS, VISA, or Cardiff), a more precise picture of the substrates that they recognize has emerged. While RIG-I binds short double-stranded RNA with triphosphate or monophosphate at the 5' end, short lengths of poly(I:C) and predominantly negative sense single stranded viral RNAs [4,5]; MDA-5 binds long lengths of poly(I:C) and mostly positive sense-single stranded viral RNA [5,6]. The RLHs trigger the production of IFN-β in response to cytoplasmic dsRNA. The RLHs contain two caspase recruitment domains (CARDs), which mediate the activation of transcription factors like NF-κB, IRF3, and IRF7. Further, IPS-1 is also a CARD-containing downstream adaptor of the RLHs. Interestingly, several cytoplasmic adaptors of death receptor signaling have been shown to be involved in RLH signaling. IPS-1 has been demonstrated to interact with Fas-associated death domain (FADD), an adaptor in Fas signaling, and receptor-interacting protein-1 (RIP1) [7]. Also, caspase-8, the apical caspase activated by tumor necrosis factor receptor (TNFR) and Fas, is cleaved in response to dsRNA, and, when over-expressed, its death effector domain (DED) can activate NF-κB in response to dsRNA [8]. Further, Michallet and coworkers reported that TNFR-associated death domain (TRADD), the proximal adaptor in TNFR signaling pathway, plays a crucial role in antiviral signaling by interacting with IPS-1 and activating NF-κB and IRF3 in response to RNA virus [9].

In view of the involvement of several death receptor proteins in RLH signaling, we investigated the role of FLIP, a key regulator of TNFR and Fas signaling in viral responses. FLIP is homologous in structure to caspase-8, but is catalytically inactive due to critical mutations in the catalytic domain [10]. By binding to TRADD or FADD and preventing caspase-8 recruitment, FLIP functions as a potent inhibitor of TNFR and Fas apoptotic signaling [11]. FLIP has also been shown to regulate NF-κB activation induced by Fas engagement [12]. Previous work from our laboratory also delineated a dual role of FLIP in response to LPS signaling through TLR4. FLIP protected against LPS-induced apoptosis and down-regulated LPS-induced NF-κB activation [13]. In this study we find that FLIP not only protected from apoptosis induced by cytoplasmic poly(I:C), a synthetic analog of dsRNA, but also down-regulated poly(I:C)-induced NF-κB and IRF3-mediated type I IFN production. Further, we find that while the caspase-8 catalytic activity is important for poly(I:C)-induced death, it was not required for IRF3-mediated gene expression.

## Methods

### Cells and reagents

Wild type MEFs, FLIP^-/+ ^heterozygous MEFs, and FLIP^-/- ^MEFs were generous gifts of Wen-Chen Yeh, Amgen Institute, Toronto, Canada. The MEFs were cultured as described in DMEM (Hyclone) enriched with 10% fetal bovine serum, glutamine (2 mmol/L), sodium pyruvate (1 mmol/L), and non essential amino acids, in the presence of penicillin (100 U/ml) and streptomycin (100 μg/ml) [13]. In our studies, wild type MEFs responded indistinguishably from FLIP^-/+ ^MEFs, and therefore either was used as a control for FLIP^-/-^MEFs. Endotoxin-free poly(I:C) was obtained from Invivogen (San Diego, CA). For poly(I:C) stimulation experiments, MEFs were transfected with 6 μg/ml of poly(I:C) using lipofectamine 2000 (LF) (Invitrogen, Carlsbad, CA) at 8 μl/ml as described [14]. LPS was obtained from Sigma-Aldrich (St. Louis, MO). Anti-FLIP monoclonal (Dave-2) and anti-β-actin polyclonal antibodies were purchased from Abcam (Cambridge, MA). Mouse IFN-β ELISA kit and the mouse IFN-β antibody were purchased from PBL Interferon Source (Piscataway, NJ). The anti-caspase-8 monoclonal antibody, 1G12, was obtained from Axxora (San Diego, CA). Sytox green nuclear stain was purchased from Invitrogen (Carlsbad, CA). Homogeneous caspase assay kit was purchased from Roche Applied Sciences (Palo Alto, CA). Alexa-488 Caspase-3 substrate was obtained from Biotium (Hayward, CA). Z-IETD-fmk and z-VAD-fmk, a caspase-8 inhibitor and a broad-spectrum caspase inhibitor, respectively, and membrane Fas ligand (mFasL) were purchased from R&D systems (Minneapolis, MN). Golgiplug™, obtained from BD Biosciences (San Jose, CA) was used at the recommended concentration (1 μl/10^6 ^cells) for 4 hours.

### Immunoblotting

MEFs were grown in 6-well dishes to a density of 10^6^/well and processed using the protocol as described [13]. Anti-FLIP antibody was used to probe the blot at a concentration of 1 μg/ml (1:1000). Afterwards, the blots were stripped using Stripping buffer (Pierce, Rockford, IL) and probed with anti β-actin antibody at 1:5000 dilution. Anti-caspase-8 and anti-IFN-β antibodies were used at 1:1000 dilution.

### RNA isolation and quantitative RT-PCR

Total RNA was extracted from the MEFs using the RNeasy RNA extraction kit from Qiagen (Germantown, MD) and cDNA synthesis was performed using 1 ug of total RNA according to the manufacturer's instructions (Invitrogen). Fluorescent quantitative real time PCR was performed using the Applied Biosystems HT 7900 system with the ABI Sybr Green PCR mastermix, and the data were analyzed using the ABI's SDS 2.1 software (Applied Biosystems, Foster City, CA). Mouse RT-PCR primer sequences are as follows: IFN-β forward primer-CCCTATGGAGATGACGGAGA and reverse primer-ACCCAGTGCTGGAGAAATTG, IFN-α4-forward primer CTGCTGGCTGTGAGGACATA and reverse primer-AGGAAGAGAGGGCTCTCCAG, IL-6 forward primer-AGTTGCCTTCTTGGGACTGA and reverse primer-TCCACGATTTCCCAGAGAAC, GAPDH forward primer-GCACAGTCAAGGCCGAGAAT and reverse primer-5'-GCCTTCTCCATGGTGGTGAA.

### Sytox green viability assay

For apoptosis assays, MEFs were seeded at a density of 10^4 ^cells/well, followed by transfection with poly(I:C) at 6 μg/ml in the presence of 8 μl LF per ml of culture medium or poly(I:C) alone at 100 μg/ml or LF alone. Membrane FasL (1 ng/ml) was used as a positive control as it is known to cause apoptosis in FLIP^-/- ^MEFs [15]. Sytox green dye (5 μM) was added simultaneously along with various treatments, and the plates were read on a Cytofluor Series 4000 fluorescence plate reader (Perseptive Biosystems Inc., Framingham, MA) at 485 nm excitation and 530 nm emission after 6 hour treatment. The fluorescence on the Y-axis is representative of cells undergoing cell death.

### Homogenous caspase assay

MEFs were seeded at 10^4 ^cells/well, cultured overnight, and either pretreated or not with z-IETD-fmk (100 μM) or z-VAD-fmk (100 μM) for 30 minutes followed by transfection with LF and poly(I:C) together, with appropriate controls such as poly(I:C), LF, or the caspase inhibitors (data not shown); and the caspase activity was measured using a fluorimetric caspase assay according to the manufacturer's instructions (Roche Applied Sciences), as described previously [13].

### Alamar Blue Viability assay

To assess viability, WT or FLIP^-/- ^MEFs were seeded into 96-well plates at a density of 20,000 cells/well. After 24 h in culture, cells were subjected to various experimental conditions and 3 h prior to the end of treatment, Alamar Blue (10% final concentration; BIOSOURCE International, Inc., Camarillo, CA) was added. Fluorescence of monolayers was assessed in a Cytofluor Series 4000 plate reader (Perseptive Biosystems Inc., Framingham, MA) at 530 nm excitation and 590 nm emission, and relative viability was expressed in arbitrary fluorescence units.

### Trypan Blue dye exclusion Viability assay

To assess viability, FLIP^-/- ^MEFs were seeded into 96-well plates at a density of 20,000 cells/well. After 24 h in culture, cells were subjected to various experimental conditions for 14 hours. Cells were washed two times with PBS, trypsinized, spun to collect, resuspended in 200 μl PBS, diluted 1:1 with Trypan blue solution, the cells that excluded the dye were counted on the hemocytometer, and the % of viable cells reported.

### Statistical analysis

Statistical analysis was performed using the Graphpad statistical package (GraphPad Software Inc., La Jolla, CA). Data are expressed as mean ± SEM, and values of p < 0.05 were considered statistically significant. A two-tailed *t *test was used to compare mean values for two-group comparisons. To compare responses between multiple groups, data were analyzed either by one-way ANOVA with Bonferroni's Multiple Comparison post hoc test or by two-way analysis of variance and the Bonferroni-post-hoc comparison test was used to compare mean values between groups.

## Results

### FLIP protects MEFs against cytoplasmic poly(I:C)-induced cell death

**Figure 1:**
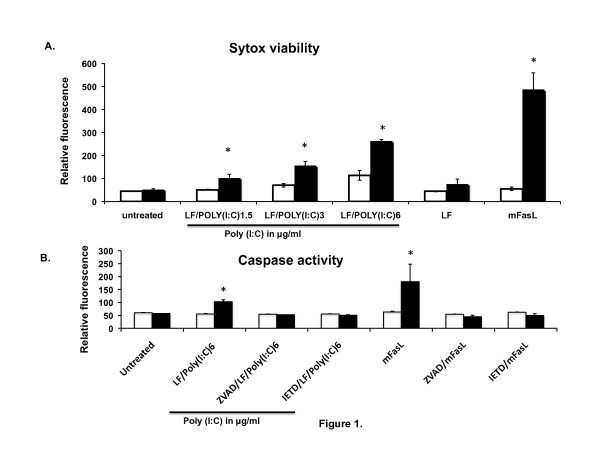
**FLIP^-/- ^MEFs are more susceptible to cytoplasmic poly(I:C)-induced killing**. (A) WT (open bars) and FLIP^-/- ^(closed bars) MEFs were untreated, transfected with LF (8 μg/ml) + poly(I:C) (1.5, 3, 6 μg/ml) or treated with mFasL (1 ng/ml) for 6 hrs and assayed for viability using the Sytox green dye. Values represent the means of 3 wells in a single experiment. Similar results were obtained in two independent experiments. *p < 0.05 compared to WT (B) WT (open bars) and FLIP^-/- ^(closed bars) MEFs were either untreated or pretreated with z-IETD-fmk or z-VAD-fmk (100 μM) for 1 hour and then treated with LF/poly(I:C) or mFasL (1 ng/ml) for 6 hrs. Viability was determined by homogeneous caspase assay. Values represent the means of 3 wells in a single experiment. *p < 0.05 compared to WT. Similar results were obtained in two independent experiments.

Wild-type and FLIP^-/- ^MEFs were assessed for their sensitivity to LF/poly(I:C) using the Sytox green assay, which is a fluorescent nuclear dye that enters only membrane compromised cells. As reported by others [14,16], we found that MEFs responded to poly(I:C) when it was transfected intracellularly by LF, but not to poly(I:C) alone (Figures 1A, B). The reason for this could be the undetectable endogenous levels of surface TLR3 in MEFs as measured by flow cytometry [data not shown and 16]. It has been previously demonstrated that TLR3 and TRIF are not involved in MEF responses to LF/poly(I:C); rather the intracellular sensors, RIG-I and IPS-1 mediate the response to cytoplasmic dsRNA [16].

FLIP^-/- ^MEFs treated with LF/poly(I:C) showed a dose-dependent increase in fluorescence, indicative of cell death, as compared to poly (I:C) (data not shown), LF, or untreated cells (Figure 1A). Compared with wild-type MEFs at 6 hours, FLIP^-/-^cells were markedly more sensitive to cell death in response to LF/poly(I:C). Also, mFasL caused massive cell death in FLIP^-/-^MEFs as compared to wild type MEFs, consistent with previous studies [15]. Previous studies have shown that intracellular dsRNA induces apoptosis by a FADD and caspase-8-dependent mechanism [17,18]. We therefore assessed caspase activation in LF/poly(I:C)-treated MEFs using a homogeneous caspase activity assay. Cells were pretreated with the broad-specificity caspase inhibitor zVAD-fmk or the caspase-8-selective inhibitor, z-IETD-fmk. LF/poly(I:C) and mFasL induced a marked increase in caspase activity in FLIP^-/-^cells compared to wild-type cells (Figure 1B), which was attenuated by pretreatment with zVAD-fmk or zIETD-fmk (Figure 1B). Further, we used the Alamar Blue metabolic assay to assess viability. Compared to WT MEFs, FLIP-/- MEFs showed a significant decline in fluorescence, indicative of cell death, in response to cytoplasmic dsRNA (Additional file 1). We also assessed cell viability by a trypan blue exclusion viability assay. As expected, treatment with LF/poly(I:C) markedly reduced viability, and this was attenuated by z-VAD-fmk (Additional file 2). Caspase activation was also detected by flow cytometric analysis using Nucview™Alexa-488-caspase 3 fluorogenic substrate, which detects caspase-3 activity in live cells. Again, FLIP^-/- ^cells showed increased fluorescence compared to wild-type MEFs, indicative of caspase-3 activity, and this was partially reversed by pretreatment with z-IETD-fmk (data not shown). These results suggest that FLIP protects MEFs from cytoplasmic dsRNA-induced caspase activation.

### FLIP suppresses LF/poly(I:C)-induced NF-κB- and IRF3-mediated gene expression in MEFs

**Figure 2:**
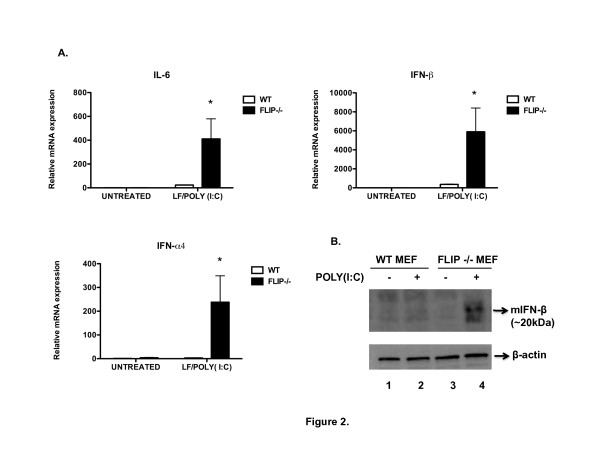
**FLIP^-/- ^MEFs show enhanced NF-κB- and IRF3-induced gene expression in response to cytoplasmic dsRNA**. A. Total RNA was isolated from WT and FLIP^-/- ^MEFs 4 hours after they were either left untreated or treated with LF (8 μg/ml) + poly(I:C) (6 μg/ml). RNA was isolated, reverse-transcribed, and mRNA levels of *il6, ifnb or ifna4 *were quantified by RT-PCR with respect to *gadph *as an endogenous control. The data are representative of three experiments with similar findings. *p < 0.05 compared to WT. B. Wild type and FLIP^-/- ^MEFs were either left untreated (lanes 1 and 3 respectively) or treated with LF/poly(I:C) (lanes 2 and 4 respectively) for 4 hrs. They were all treated with Brefeldin A (Golgiplug™) for 4 hours. Cell lysates were prepared and analyzed by immunoblot for IFN-β. The data are representative of two experiments with similar results.

Using wild type and FLIP-/- MEFs, we examined gene expression in response to intracellular dsRNA. The expression of *ifna*4 is regulated by IRF3, *il-6 *by NF-κB, and *ifnb *by the cooperative activation of both transcription factors [19-21]. Wild-type cells showed increased expression of *il-6*, *ifna4*, and *ifnb *genes after 4 hrs of stimulation with LF/poly(I:C) as compared to untreated cells. Notably, compared to wild type MEFs, FLIP^-/-^MEFs showed markedly increased induction for all 3 genes (Figure 2A).Untreated cells or cells treated with LF or poly(I:C) alone did not induce significant gene expression in either wild-type or FLIP^-/-^MEFs (data not shown).

In order to confirm results obtained by mRNA expression, IFN-β protein was assessed by ELISA. In response to LF/poly(I:C), FLIP^-/-^MEFs generated more IFN-β than wild-type cells or FLIP^-/-^cells reconstituted with FLIP (data not shown), and this overproduction was unaffected when FLIP^-/-^cells were pretreated with the caspase 8 inhibitor to prevent cell death during the incubation (data not shown). This result suggests that caspase-8 catalytic activity is not involved in generation of IFN-β in response to cytoplasmic dsRNA (vide infra).

We also examined levels of intracellular IFN-β in MEFs treated with Golgiplug™ (brefeldin A), which prevents cytokine secretion [22]. Compared to WT MEFs, FLIP^-/- ^cells showed increased intracellular levels of IFN-β in response to LF/poly(I:C) (Figure 2B).

### Reconstitution of FLIP reverses the enhanced NF-KB and IRF3 activation in FLIP^-/- ^MEFs in response to cytoplasmic dsRNA

**Figure 3:**
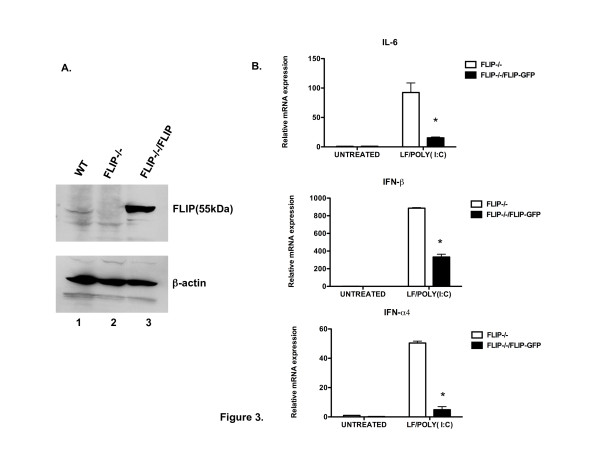
**Reconstitution of FLIP reverses the enhanced NF-KB and IRF3 gene expression in FLIP^-/- ^cells in response to cytoplasmic poly(I:C)**. (A). FLIP^+/- ^(lane1), FLIP^-/- ^(lane 2), and reconstituted FLIP^-/- ^(lane 3) MEFs were subjected to immunoblot analysis with anti-FLIP antibody. (B). FLIP^-/- ^MEFs stably transfected with GFP vector alone or with a vector carrying FLIP-GFP were left untreated, serving as untreated control, or treated with LF/poly(I:C) for 4 hrs and analyzed by RT-PCR for expression of *il-6, ifnb *or *ifna*4 relative to *gapdh*. *p < 0.05, compared to FLIP-/- MEFs carrying GFP vector. The data are representative of three experiments with similar results.

In order to confirm that the enhanced NF-κB- and IRF3-mediated gene expression noted above (Figure 2A,B) was due to the absence of FLIP, we tested FLIP^-/-^MEFs, which had been stably transfected with a GFP vector alone or vector harboring full-length FLIP [13]. Western blot analysis confirmed the over-expression of FLIP in FLIP^-/-^MEFs that were reconstituted with FLIP (Figure 3A). Upon stimulation with LF/poly(I:C), FLIP^-/-^MEFs reconstituted with GFP vector alone showed increased expression of *il-6*, *ifna4*, and *ifnb *genes compared to cells reconstituted with FLIP (Figure 3B). Thus, the reconstitution of FLIP in the FLIP^-/- ^cells reversed the enhancement of NF-κB- and IRF3-induced gene activation in response to cytoplasmic dsRNA.

### Caspase-8 catalytic activity is not required for intracellular dsRNA-mediated signaling

**Figure 4:**
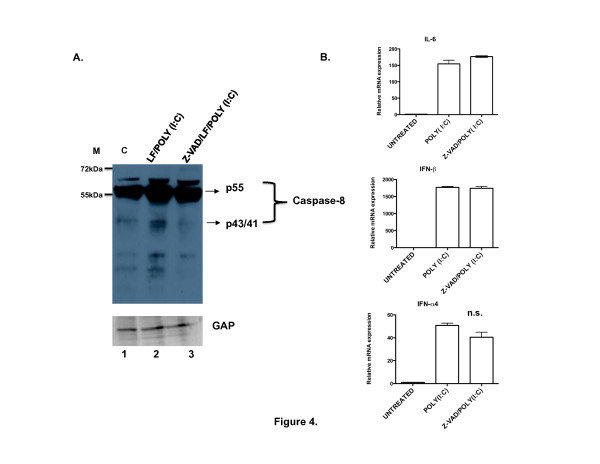
**Caspase-8 proteolytic activity is not required for cytoplasmic dsRNA-induced signaling**. (A). FLIP^-/- ^MEFs were left untreated (lane 1), or treated with LF/poly(I:C) for 2 hours (lane 2), or pretreated for 1 hr with z-VAD-fmk (100 μM) followed by treatment with LF/poly(I:C) for 2 hours (lane 3). The cell lysates were then analyzed for procaspase-8 processing by immunoblot. (B). FLIP^-/- ^MEFs were either left untreated or pretreated for 1 hr with z-VAD-fmk (100 μM) followed by LF/poly(I:C). After 4 hours the cells were analyzed by RT-PCR for expression of *il-6, ifnb *or *ifna4 *relative to *gapdh*. The data are representative of three experiments with similar findings.

Takahashi and others [8] reported that caspase-8 processing occurs in response to dsRNA stimulation and that the caspase-8-deficient MEFs have impaired NF-κB-mediated gene expression but intact IRF3 signaling. Based on these observations, we investigated whether caspase-8 catalytic activity was required for NF-κB and IRF3-induced gene expression in response to cytoplasmic dsRNA in FLIP-/-MEFs. We first confirmed that caspase-8 was activated in the FLIP^-/-^cells by immunoblot analysis of procaspase-8 proteolytic cleavage in response to LF/poly(I:C). As shown in Figure 4A, FLIP^-/-^cells showed increased caspase-8 processing as indicated by the appearance of lower molecular weight cleavage products upon treatment with LF/poly(I:C) compared to untreated FLIP^-/-^cells. This processing was reduced by z-VAD-fmk pretreatment. We also confirmed that treatment with mFasL led to caspase-8 cleavage, which was reduced by pretreatment with z-VAD-fmk (Additional file 3). Having demonstrated that procaspase-8 is processed in FLIP^-/-^cells treated with LF/poly(I:C), similar to mFasL, and that processing was attenuated with a caspase-selective inhibitor, we next examined the effect of inhibition of caspase-8 catalytic activity on NF-κB- and IRF3-induced gene expression. FLIP^-/-^cells were pretreated with the z-VAD-fmk, for 1 hour followed by incubation with LF/poly(I:C) for 4 hours. Cells were analyzed for the expression of NF-κB and IRF3 target genes by RT-PCR. We found that z-VAD-fmk did not significantly affect the level of induction of *il-6, ifnb *or *ifna4* (Figure 4B), suggesting that caspase-8 catalytic activity was not required NF-κB- and IRF3-induced gene expression.

## Discussion

There is compelling evidence that the death receptor signaling proteins TRADD, FADD, and caspase-8 are important components of cellular responses to cytoplasmic dsRNA. Michallet *et al *showed that TRADD is an essential component of the RIG-like helicase antiviral pathway [9]. Their studies showed that TRADD, a crucial adaptor of the TNFRI, was recruited by IPS-1 and regulated complex formation with TRAF3/TANK and FADD/RIP1, leading to the activation of IRF3 and NF-κB. Balachandran *et al *[14] and Takahashi *et al *[8] showed that FADD is required for both IRF3- and NF-κB-mediated activation in response to poly(I:C). Kawai *et al *[7] further demonstrated that FADD and RIP1 interact with IPS-1.

We now show that the caspase-8 homologue FLIP is a negative regulator of cytoplasmic dsRNA-induced activation of IRF3 and NF-κB. FLIP is homologous in structure to caspase-8, but is catalytically inactive due to critical mutations in the catalytic domain [13]. FLIP associates with FADD by homophilic interaction of the death effector domains (DED) contained within each molecule [13]. One explanation for our results is that in the absence of FLIP there is increased availability of FADD for formation of the RLH signaling module of TRADD-FADD-RIP1 [9], thus accounting for the increased activation NF-κB and IRF3 in FLIP^-/- ^cells. Additionally, FLIP, via its DED, interacts with NF-kappaB-inducing kinase (NIK) and IkappaB kinase (IKK) [13]. Since these molecules are involved in the dsRNA induction of NF-κB activation [23], FLIP binding and/or sequestering of NIK and IKK could hinder their ability to promote NF-κB signaling.

Our studies also show that caspase activity may not be required for activation of IRF3 and NF-κB in response to intracellular dsRNA. Using z-VAD-fmk at doses (100 μM) that abrogated all intracellular caspase activity induced by LF/poly(I:C), we found no effect of caspase blockade on LF/poly(I:C)-induced IRF3-and NF-κB-mediated gene expression. These results are in agreement with Balachandran *et al *[14] who showed that activation of an IFN-β promoter by LF/poly(I:C) was similar in caspase-8^-/-^and WT MEFs. Furthermore, in their studies, although z-VAD-fmk (100 μM) prevented apoptosis induced by a Fas agonist antibody, the caspase inhibitor did not impair LF/poly(I:C)-induced IFN-α production [14]. Takahashi *et al *[8] showed that LF/poly(I:C)-induced IRF3 activation was unaffected and early NF-κB activation was reduced in caspase-8^-/- ^MEFs. Most recently, Rajput *et al*, reported that there was enhanced activation of IRF3 by poly(I:C) in caspase-8^-/- ^MEFs and that RIG-I-mediated activation of IRF-3 induced by intracellular dsRNA was negatively regulated by a caspase-8-dependent cleavage of RIP1 protein [24]. They further reported that siRNA-mediated knock-down of caspase-8 had no effect on NF-κB activation by Sendai virus infection. Together, it appears that caspase-8 activity may not be required for IRF3 or NF-κB activation induced by cytoplasmic dsRNA.

## Conclusions

In conclusion, we demonstrate a pivotal role for FLIP in protecting cells from the apoptosis and attenuating cytokine responses induced by cytoplasmic dsRNA. We have identified FLIP as a crucial molecule that regulates the response in MEFs: FLIP suppresses both inflammatory and apoptotic pathways. Strategies to increase expression of FLIP would be expected to dampen the excessive inflammation as well as apoptosis.

## Competing interests

The authors declare that they have no competing interests.

## Authors' contributions

All authors have read and approved the manuscript.

Conceived and designed the experiments: PH, JH. Performed the experiments: PH, JT, KJ. Analyzed the data: PH, JH. Wrote the paper: PH, JH.

## Acknowledgements

We would like to acknowledge the financial support from NIH grant GM071398 (JH).

## Supplementary Material

Additional file 1 **Viability of WT and FLIP^-/- ^MEFs as assessed by Alamar Blue assay is reduced by treatment with LF/poly(I:C)**. Metabolic activity of WT and FLIP^-/- ^MEFs was assessed by Alamar Blue assay after 14 hours of treatment with medium alone, LF (8 μl), poly(I:C) (6 μg/ml), or LF/poly(I:C). Values represent means (± S.E.) with metabolic activity reported in arbitrary fluorescent units. p < 0.05, *, LF/poly(I:C) significantly reduced viability compared to untreated FLIP-/- MEFs. Click here for file

Additional file 2 **Viability of FLIP^-/- ^MEFs as assessed by Trypan Blue dye exclusion is reduced by treatment with LF/poly(I:C)**. Percent of FLIP^-/- ^MEF cells excluding Trypan Blue dye was assessed after 14 hours treatment with medium alone, dimethyl sulfoxide (DMSO), LF (8 μl), poly(I:C) (6 μg/ml), or LF/poly(I:C) with or without z-VAD-fmk (10 or 100 μM). p < 0.05, *, LF/poly(I:C) significantly reduced viability compared to untreated cells, #, p < 0.05, 100 μM z-VAD-fmk significantly inhibits LF/poly(I:C) induced cell death. Click here for file

Additional file 3 **Treatment with mFasL induces processing of caspase-8 in FLIP^-/- ^MEFs**. FLIP^-/- ^MEFs were left untreated (lane 1), or treated with mFasL for 6 hours (lane 2), or pretreated for 1 hr with z-VAD-fmk (100 μM) followed by treatment with mFasL for 6 hours (lane 3). The cell lysates were then analyzed for procaspase-8 processing by immunoblot. Click here for file

## References

[B1] Kawai T Akira S Toll-like receptor and RIG-I-like receptor signaling Ann N Y Acad Sci 2008 1143 1 20 10.1196/annals.1443.020 19076341

[B2] Takeda K Akira S Toll-like receptors in innate immunity Int Immunol 2005 17 1 14 15585605 10.1093/intimm/dxh186

[B3] Meylan E Tschopp J Toll-like receptors and RNA helicases: two parallel ways to trigger antiviral responses Mol Cell 2006 22 561 569 10.1016/j.molcel.2006.05.012 16762830

[B4] Schlee M Roth A Hornung V Hagmann CA Wimmenauer V Barchet W Coch C Janke M Mihailovic A Wardle G Juranek S Kato H Kawai T Poeck H Fitzgerald KA Takeuchi O Akira S Tuschl T Latz E Ludwig J Hartmann G Recognition of 5' triphosphate by RIG-I helicase requires short blunt double-stranded RNA as contained in panhandle of negative-strand virus Immunity 2009 31 25 34 10.1016/j.immuni.2009.05.008 19576794 PMC2824854

[B5] Kumar H Kawai T Akira S Pathogen recognition by the innate immune system Int Rev Immunol 2011 30 16 34 10.3109/08830185.2010.529976 21235323

[B6] Kato H Takeuchi O Sato S Yoneyama M Yamamoto M Matsui K Uematsu S Jung A Kawai T Ishii KJ Yamaguchi O Otsu K Tsujimura T Koh CS Reis e Sousa C Matsuura Y Fujita T Akira S Differential roles of MDA5 and RIG-I helicases in the recognition of RNA viruses Nature 2006 441 101 5 10.1038/nature04734 16625202

[B7] Takahashi K Sato S Coban C Kumar H Kato H Ishii KJ Takeuchi O Akira S IPS-1, an adaptor triggering RIG-I- and Mda5-mediated type I interferon induction Nat Immunol 2005 6 981 988 10.1038/ni1243 16127453

[B8] Takahashi K Kawai T Kumar H Sato S Yonehara S Akira S Roles of caspase-8 and caspase-10 in innate immune responses to double-stranded RNA J Immunol 2006 176 4520 4524 16585540 10.4049/jimmunol.176.8.4520

[B9] Michallet MC Meylan E Ermolaeva MA Vazquez J Rebsamen M Curran J Poeck H Bscheider M Hartmann G König M Kalinke U Pasparakis M Tschopp J TRADD protein is an essential component of the RIG-like helicase antiviral pathway Immunity 2008 28 651 61 10.1016/j.immuni.2008.03.013 18439848

[B10] Krueger A Baumann S Krammer PH Kirchhoff S FLICE-inhibitory proteins: regulators of death receptor-mediated apoptosis Mol Cell Biol 2001 21 8247 8254 10.1128/MCB.21.24.8247-8254.2001 11713262 PMC99990

[B11] Hughes MA Harper N Butterworth M Cain K Cohen GM MacFarlane M Reconstitution of the death-inducing signaling complex reveals a substrate switch that determines CD95-mediated death or survival Mol Cell 2009 35 265 79 10.1016/j.molcel.2009.06.012 19683492

[B12] Kreuz S Siegmund D Rumpf JJ Samel D Leverkus M Janssen O Häcker G Dittrich-Breiholz O Kracht M Scheurich P Wajant H NFkappaB activation by Fas is mediated through FADD, caspase-8, and RIP and is inhibited by FLIP J Cell Biol 2004 166 369 80 10.1083/jcb.200401036 15289496 PMC2172264

[B13] Bannerman DD Eiting KT Winn RK Harlan JM FLICE-like inhibitory protein (FLIP) protects against apoptosis and suppresses NF-kappaB activation induced by bacterial lipopolysaccharide Am J Pathol 2004 165 1423 1431 10.1016/S0002-9440(10)63400-1 15466406 PMC1618633

[B14] Balachandran S Thomas E Barber GN A FADD-dependent innate immune mechanism in mammalian cells Nature 2004 432 401 405 10.1038/nature03124 15549108

[B15] Yeh WC Itie A Elia AJ Ng M Shu HB Wakeham A Mirtsos C Suzuki N Bonnard M Goeddel DV Mak TW Requirement for Casper (c-FLIP) in regulation of death receptor-induced apoptosis and embryonic development Immunity 2000 12 633 42 10.1016/S1074-7613(00)80214-9 10894163

[B16] Balachandran S Venkataraman T Fisher PB Barber GN Fas-associated death domain-containing protein-mediated antiviral innate immune signaling involves the regulation of Irf7 J Immunol 2007 178 2429 39 17277150 10.4049/jimmunol.178.4.2429

[B17] Balachandran S Kim CN Yeh WC Mak TW Bhalla K Barber GN Activation of the dsRNA-dependent protein kinase, PKR, induces apoptosis through FADD-mediated death signaling EMBO J 1998 17 6888 902 10.1093/emboj/17.23.6888 9843495 PMC1171037

[B18] Iordanov MS Kirsch JD Ryabinina OP Wong J Spitz PN Korcheva VB Thorburn A Magun BE Recruitment of TRADD, FADD, and caspase 8 to double-stranded RNA-triggered death inducing signaling complexes (dsRNA-DISCs) Apoptosis 2005 10 167 76 10.1007/s10495-005-6071-x 15711932

[B19] Bonjardim CA Interferons (IFNs) are key cytokines in both innate and adaptive antiviral immune responses and viruses counteract IFN action Microbes Infect 2005 7 569 78 10.1016/j.micinf.2005.02.001 15792636

[B20] Andersen J VanScoy S Cheng TF Gomez D Reich NC IRF-3-dependent and augmented target genes during viral infection Genes Immun 2008 9 168 75 10.1038/sj.gene.6364449 18094709

[B21] Miettinen M Sareneva T Julkunen I Matikainen S IFNs activate toll-like receptor gene expression in viral infections Genes Immun 2001 2 349 55 10.1038/sj.gene.6363791 11607792

[B22] Reimer T Brcic M Schweizer M Jungi TW Poly(I:C) and LPS induce distinct IRF3 and NF-kappaB signaling during type-I IFN and TNF responses in human macrophages J Leukoc Biol 2008 83 1249 57 10.1189/jlb.0607412 18252870

[B23] Zamanian-Daryoush M Mogensen TH DiDonato JA Williams BR NF-kappaB activation by double-stranded-RNA-activated protein kinase (PKR) is mediated through NF-kappaB-inducing kinase and IkappaB kinase Mol Cell Biol 2000 20 4 1278 90 10.1128/MCB.20.4.1278-1290.2000 10648614 PMC85265

[B24] Rajput A Kovalenko A Bogdanov K Yang SH Kang TB Kim JC Du J Wallach D RIG-I RNA Helicase Activation of IRF3 Transcription Factor Is Negatively Regulated by Caspase-8-Mediated Cleavage of the RIP1 Protein Immunity 2011 34 340 51 10.1016/j.immuni.2010.12.018 21419663

