# Supplementary material for: FLIP (Flice-like inhibitory protein) suppresses cytoplasmic double-stranded-RNA-induced apoptosis and NF-κB and IRF3-mediated signaling
Source: Cell Commun Signal. 2011 Jun 2;9:16. doi: 10.1186/1478-811X-9-16 (PMC3129316; doi:10.1186/1478-811X-9-16)
Supplement: Additional file 1 — Viability of WT and FLIP-/- MEFs as assessed by Alamar Blue assay is reduced by treatment with LF/poly(I:C). Metabolic activity of WT and FLIP-/- MEFs was assessed by Alamar Blue assay after 14 hours of treatment with medium alone, LF (8 μl), poly(I:C) (6 μg/ml), or LF/poly(I:C). Values represent means (± S.E.) with metabolic activity reported in arbitrary fluorescent units. p < 0.05, *, LF/poly(I:C) significantly reduced viability compared to untreated FLIP-/- MEFs. [file 1478-811X-9-16-S1.PDF]

Alamar Blue fluorescence

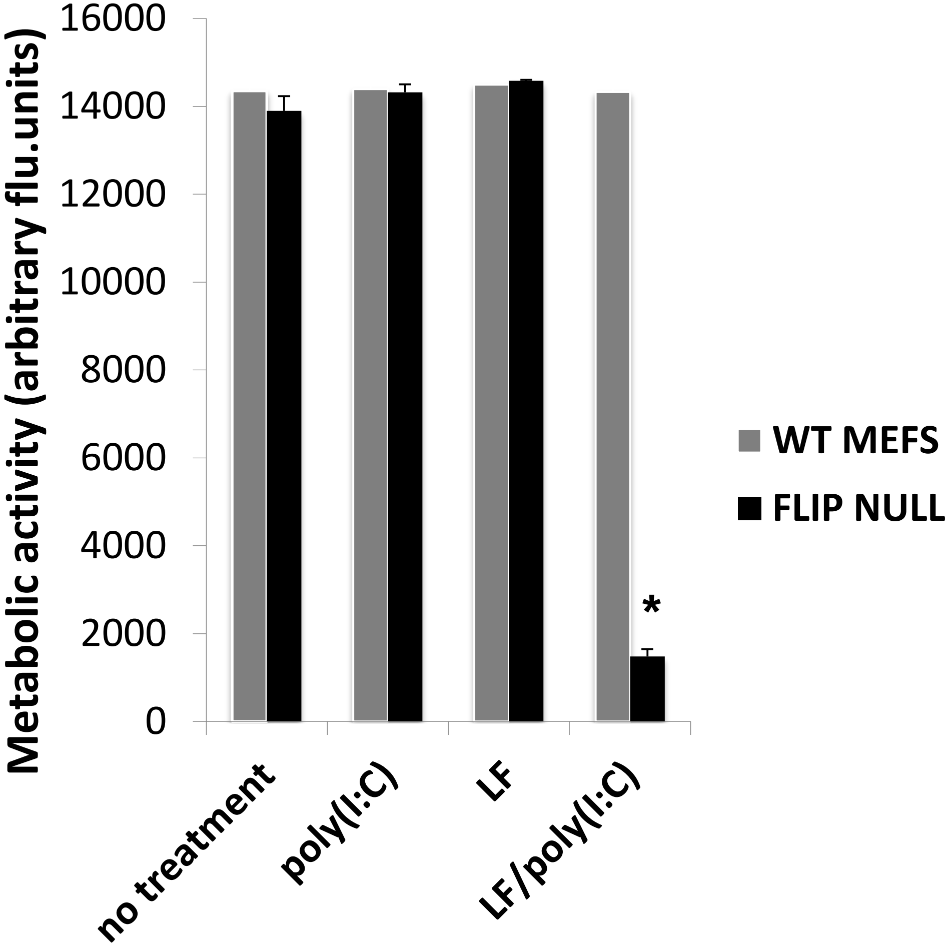

Additional file 1. Viability of WT and FLIP-/- MEFs as assessed by Alamar Blue assay is reduced by treatment with LF/poly(I:C). Metabolic activity of WT and FLIP-/- MEFs was assessed by Alamar Blue assay after 14 hours treatment with medium alone, LF (8 μl), poly(I:C) (6 μg/ml) , or LF/poly(I:C). Values represent means (± S.E.) with metabolic activity reported in arbitrary fluorescent units. $p<0.05$, *, LF/poly(I:C) significantly reduced viability compared to untreated FLIP-/- MEFs.

Additional file 1
