# Supplementary material for: FLIP (Flice-like inhibitory protein) suppresses cytoplasmic double-stranded-RNA-induced apoptosis and NF-κB and IRF3-mediated signaling
Source: Cell Commun Signal. 2011 Jun 2;9:16. doi: 10.1186/1478-811X-9-16 (PMC3129316; doi:10.1186/1478-811X-9-16)
Supplement: Additional file 2 — Viability of FLIP-/- MEFs as assessed by Trypan Blue dye exclusion is reduced by treatment with LF/poly(I:C). Percent of FLIP-/- MEF cells excluding Trypan Blue dye was assessed after 14 hours treatment with medium alone, dimethyl sulfoxide (DMSO), LF (8 μl), poly(I:C) (6 μg/ml), or LF/poly(I:C) with or without z-VAD-fmk (10 or 100 μM). p < 0.05, *, LF/poly(I:C) significantly reduced viability compared to untreated cells, #, p < 0.05, 100 μM z-VAD-fmk significantly inhibits LF/poly(I:C) induced cell death. [file 1478-811X-9-16-S2.PDF]

Trypan Blue Viability assay
FLIP-/- MEFs

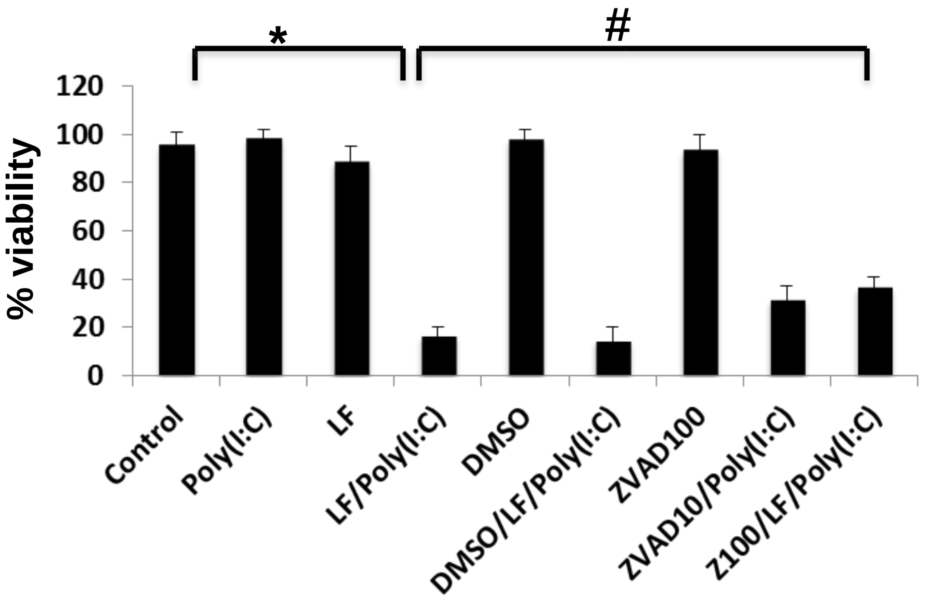

Additional file 2. Viability of FLIP$^{-/-}$ MEFs as assessed by Trypan Blue dye exclusion is reduced by treatment with LF/poly(I:C). Percent of FLIP$^{-/-}$ MEF cells excluding Trypan Blue dye was assessed after 14 hours treatment with medium alone, dimethyl sulfoxide (DMSO), LF (8 μl), poly(I:C) (6 μg/ml), or LF/poly(I:C) with or without z-VAD-fmk (10 or 100 μM). $p<0.05$, *, LF/poly(I:C) significantly reduced viability compared to untreated cells , #, $p<0.05$, 100 μM z-VAD-fmk significantly inhibits LF/poly(I:C) induced cell death.

Additional file 2
