# Supplementary material for: FLIP (Flice-like inhibitory protein) suppresses cytoplasmic double-stranded-RNA-induced apoptosis and NF-κB and IRF3-mediated signaling
Source: Cell Commun Signal. 2011 Jun 2;9:16. doi: 10.1186/1478-811X-9-16 (PMC3129316; doi:10.1186/1478-811X-9-16)
Supplement: Additional file 3 — Treatment with mFasL induces processing of caspase-8 in FLIP-/- MEFs. FLIP-/- MEFs were left untreated (lane 1), or treated with mFasL for 6 hours (lane 2), or pretreated for 1 hr with z-VAD-fmk (100 μM) followed by treatment with mFasL for 6 hours (lane 3). The cell lysates were then analyzed for procaspase-8 processing by immunoblot. [file 1478-811X-9-16-S3.PDF]

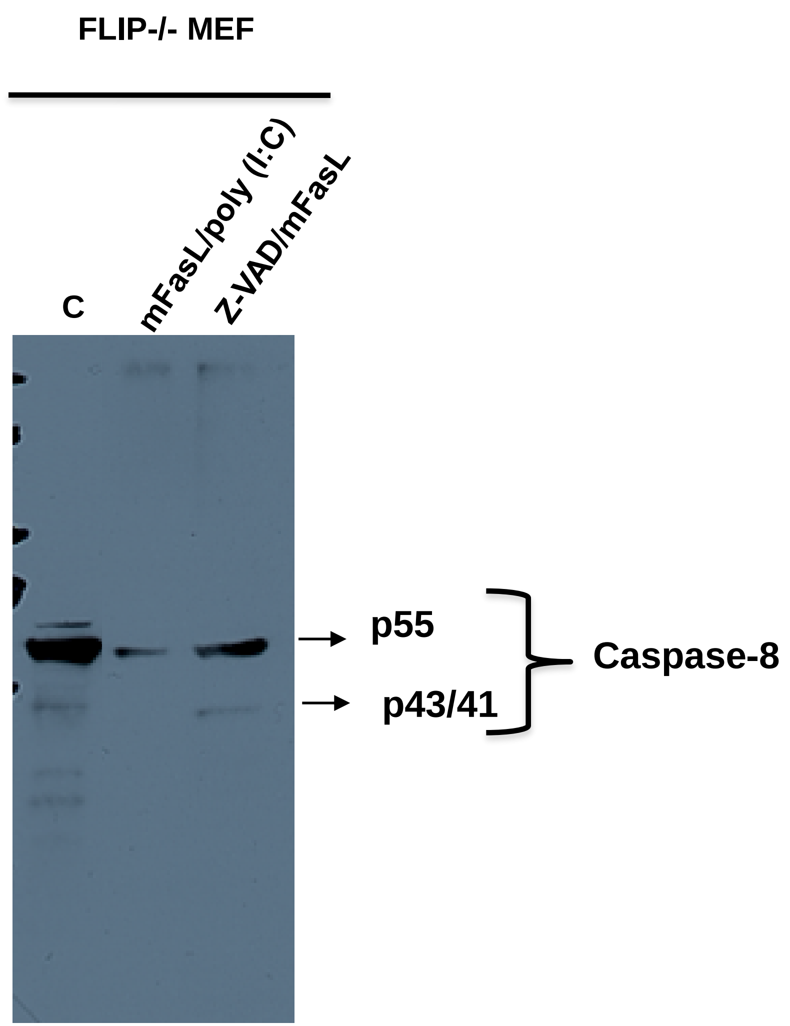

Additional file 3. Treatment with mFasL induces processing of caspase-8 in FLIP$^{-/-}$ MEFs. FLIP$^{-/-}$ MEFs were left untreated (lane 1), or treated with mFasL for 6 hours (lane 2), or pretreated for 1 hr with z-VAD-fmk (100 μM) followed by treatment with mFasL for 6 hours (lane 3). The cell lysates were then analyzed for procaspase-8 processing by immunoblot.

Additional file 3
